# Organ‐level validation of a cross‐bridge cycling descriptor in a left ventricular finite element model: effects of ventricular loading on myocardial strains

**DOI:** 10.14814/phy2.13392

**Published:** 2017-11-09

**Authors:** Sheikh Mohammad Shavik, Samuel T. Wall, Joakim Sundnes, Daniel Burkhoff, Lik Chuan Lee

**Affiliations:** ^1^ Department of Mechanical Engineering Michigan State University East Lansing Michigan; ^2^ Simula Research Laboratory Oslo Norway; ^3^ Cardiovascular Research Foundation and Department of Medicine Columbia University New York New York

**Keywords:** Cardiac energetics, finite element modeling, left ventricle, myocardial strain

## Abstract

Although detailed cell‐based descriptors of cross‐bridge cycling have been applied in finite element (FE) heart models to describe ventricular mechanics, these multiscale models have never been tested rigorously to determine if these descriptors, when scaled up to the organ‐level, are able to reproduce well‐established organ‐level physiological behaviors. To address this void, we here validate a left ventricular (LV) FE model that is driven by a cell‐based cross‐bridge cycling descriptor against key organ‐level heart physiology. The LV FE model was coupled to a closed‐loop lumped parameter circulatory model to simulate different ventricular loading conditions (preload and afterload) and contractilities. We show that our model is able to reproduce a linear end‐systolic pressure volume relationship, a curvilinear end‐diastolic pressure volume relationship and a linear relationship between myocardial oxygen consumption and pressure**–**volume area. We also show that the validated model can predict realistic LV strain‐time profiles in the longitudinal, circumferential, and radial directions. The predicted strain‐time profiles display key features that are consistent with those measured in humans, such as having similar peak strains, time‐to‐peak‐strain, and a rapid change in strain during atrial contraction at late‐diastole. Our model shows that the myocardial strains are sensitive to not only LV contractility, but also to the LV loading conditions, especially to a change in afterload. This result suggests that caution must be exercised when associating changes in myocardial strain with changes in LV contractility. The methodically validated multiscale model will be used in future studies to understand human heart diseases.

## Introduction

Finite element (FE) modeling of the intact heart having realistic geometry and architectural assembly of cardiac muscle descriptor has advanced significantly over the years. Such models are now capable of describing the coupling between electrophysiology and mechanics (Kerckhoffs et al. 2010; Gurev et al. 2011; Sundnes et al. 2014), as well as long‐term remodeling of the heart (Göktepe et al. 2010; Kerckhoffs et al. 2012; Lee et al. 2015, 2016). Increasingly, computer models are also used to elucidate the pathophysiology of heart disease and mechanisms of treatments (e.g., cardiac resynchronization therapy (Hu et al. 2014; Kerckhoffs et al. 2009) and surgical ventricular restoration (Lee et al. 2013). Accurate description of active contraction behavior is particularly important in ensuring that the model predictions are consistent with well‐established physiological principles at the whole heart level.

Available contraction models vary in sophistication and detail depending on the number of intermediate cross‐bridge states used in dynamically modeling muscle fiber shortening. Such descriptions range from being purely phenomenological (Guccione and McCulloch 1993; Kerckhoffs et al. 2007) that do not model the different states of the cross‐bridge cycle, to detailed models (Burkhoff 1994; Hunter et al. 1998; Rice et al. 2008) that describe some (if not all) the states of the cross‐bridge cycle. For the latter case, models were usually developed based on small‐sample measurements of force‐calcium and force‐velocity relationships under different loading conditions using multicellular (papillary or trabecular muscles), intact single‐cell or skinned fiber preparations. Although able to recapitulate key features found in these small‐scale measurements, it is unknown if these descriptors, when scaled up with realistic ventricular geometry and muscle fiber organization in the ventricular wall, can reproduce key features of measurements made at the whole organ level. Correspondingly, there exists a question as to whether the gap between subcellular and tissue‐organ level phenomena can be bridged by simply applying these detailed cross‐bridge models to describe the mechanical behavior of the whole heart.

Here, we aim to validate, in a rigorous manner, a left ventricular FE computer model that is based on a detailed descriptor of cross‐bridge cycling by Rice et al. (2008) against well‐established features of organ‐level physiology. Originally calibrated using experimental data from rats, this model has been adopted in many multiscale computational frameworks used to investigate diseases and treatments in relation to data acquired from other species, such as humans (Adeniran et al. 2013a, 2013b, 2015; Hu et al. 2013) and canine (Gurev et al. 2011; Constantino et al. 2012; Lim et al. 2012; Hu et al. 2014). While model predictions using this cross‐bridge descriptor have been compared to some myocardial strain and single pressure**–**volume (PV) loop measurements (Campbell et al. 2008; Gurev et al. 2011; Trayanova et al. 2011), there are no existing studies investigating whether the model can reproduce features found in an analysis of human heart behavior in the face of varying preload and afterload, such as a linear, relatively load‐independent end‐systolic pressure**–**volume relationship (ESPVR). It is also not known whether the Rice cross‐bridge descriptor, when scaled up, is able to reproduce the experimentally observed linear relationship between total myocardial oxygen consumption (MVO_2_) and total mechanical work (indexed by the pressure**–**volume area, PVA). It is the purpose of this study to address these voids in order to develop a model that can be useful for studying the complex relationship between myocyte contractility and whole heart function, which depends on multiple factors such as the fiber orientation, muscle mass, heart geometry, and activation sequence.

## Method

### Mechanical description of the left ventricle

The mechanical behavior of the left ventricle (LV) was modeled using a previously described cell‐based coupled cardiac electromechanics model (Sundnes et al. 2014). Given that the focus here is on the mechanics, homogeneous activation of the LV was simulated by applying a stimulus current *I_s_* throughout the ventricle. Consequently, the resultant model can be expressed by the following system of ordinary differential equations and partial differential equations:

(1a) $$\frac{\partial s}{\partial t}=f\left(v,s,\lambda \right)$$

(1b) $$\frac{\partial v}{\partial t}+I_{\mathrm{ion}}\left(v,s,\lambda \right)=I_{s}$$

(1c) ∇·σ=0

Equations (1a and b) consist of a system of ordinary differential equations describing the local coupling between the cellular electrophysiology (Winslow et al. 1999) and cross‐bridge cycling (Rice et al. 2008). Here, *v* is the transmembrane potential, **s** denotes a vector of state variables consisting of various membrane channels and intracellular ionic concentrations, *I*_ion_ is the total ionic current that is scaled with the membrane capacitance, and *λ* is the myofiber stretch. Equation (1c) enforces local mechanical equilibrium of the LV with ***σ*** denoting the Cauchy stress tensor. The stress tensor ***σ*** was additively decomposed into a passive component ***σ***_**p**_ and an active component ***σ***_**a**_, allowing for dynamic changes in the tissue during the cross‐bridge cycling process, i.e.,

(2) $$\sigma =\sigma_{p}+\sigma_{a}\left(s,\lambda ,\dot{\lambda };T_{\mathrm{ref}}\right)$$

The active stress ***σ***_**a**_, applied along the local fiber orientation, was based on the active descriptor by Rice et al. (2008), and depends on the time evolution of the state variables **s**, myofiber stretch *λ*, rate of myofiber stretch $\dot{\lambda }$ and the reference tension *T*_ref_. Parameter values of the Rice descriptor were modified to obtain a twitch profile with a longer time to peak duration and a reasonable relaxation behavior (cf. to that computed using the original values) that are both necessary to reproduce the in‐vivo strain‐time profile measured in healthy humans. These parameters are associated to the Ca‐binding with troponin (Ca‐based activation) and transition rate between different states in the cross‐bridge cycle. Some of these parameters were also adjusted in a previous study (Provost et al. 2011) to reproduce end‐systolic pressure and pressure twitch recorded experimentally in the canine. The modified parameters are tabulated in Table 1. Contractility was varied by scaling the calcium transient through adjusting the maximum RyR channel Ca‐flux $v_{1}$, scaling factor of Ca‐ATPase $K_{SR}$, and maximum sarcolemmal Ca‐pump current ${\overset{¯}{I}}_{p_{\left(Ca\right)}}$ in the electrophysiology model (Winslow et al. 1999) as listed in Table 2. Modified parameters of the electrophysiology model (Table 2) reflect the corresponding changes in the isometric twitch profiles (Fig. 2) for different contractility.

**Table 1:** Modified parameters of the Rice model (Rice et al. 2008)

Parameter	Unit	Value
perm_50_	Unitless	0.38
*n*_perm_	Unitless	13
*k*_on_	*μ*mol L^−1^ s^−1^	60
*k*_n_p_	s^−1^	15
*k*_p_n_	s^−1^	550
*g*_app_	s^−1^	10
*h*_f_	s^−1^	750
*h*_b_	s^−1^	70
*g*_xb_	s^−1^	20

**Table 2:** Modified parameters of the cellular electrophysiology model (Winslow et al. 1999) that alters the calcium transient to produce a change in contractility

Parameter	Unit	Baseline contractility	Lower contractility	Higher contractility
*V*_1_	msec^−1^	1.8	1.2	4.2
*K*_SR_	unitless	1.3	0.7	1.4
${\overset{¯}{I}}_{p_{\left(Ca\right)}}$	*μ*A*μ*F^−1^	0.05	0.3	0.008

On the other hand, the passive stress ***σ***_**p**_ was described using a Fung‐type transversely isotropic hyperelastic constitutive model (Guccione et al. 1991) with the strain energy function given by

(3a) $$\Psi =\frac{1}{2}C\left(e^{Q}-1\right)+C_{\mathrm{compr}}\left(J\text{ln}J-J+1\right)$$

where,

(3b) $$\begin{array}{cc} Q & =b_{\mathrm{ff}}E_{\mathrm{ff}}^{2}+b_{\mathrm{xx}}\left(E_{\mathrm{ss}}^{2}+E_{\mathrm{nn}}^{2}+E_{\mathrm{sn}}^{2}+E_{\mathrm{ns}}^{2}\right)\\ & +b_{\mathrm{fx}}\left(E_{\mathrm{fn}}^{2}+E_{\mathrm{nf}}^{2}+E_{\mathrm{fs}}^{2}+E_{\mathrm{sf}}^{2}\right) \end{array}$$

In the above equation, *E*_*ij*_ with (*i, j*)* *∈ (*f, s, n*) are components of the Green‐Lagrange strain tensor ***E*** with *f*,* s*,* n* denoting the myocardial fiber, sheet and sheet normal directions respectively. Furthermore, *J *= det (**F**) is the Jacobian of the deformation gradient tensor **F**. Material parameters of the passive constitutive model are denoted by *C*_compr_, *C*,* b*_ff_, *b*_xx_, and *b*_*f*x_. The passive stress tensor depends on this strain energy function by

(4) $$\sigma_{p}=\frac{1}{J}F\frac{\partial \Psi }{\partial E}F^{T}$$

The governing equations describing the LV mechanical behavior were solved using the FE method.

An idealized prolate ellipsoid was used to describe the LV geometry, which was discretized using 960 quadratic tetrahedral elements. The LV base was constrained from moving out of the plane and the epicardial edge was fixed (Wenk et al. 2012; Genet et al. 2014). Based on previous experimental measurements (Streeter et al. 1969), myofiber helix angle was prescribed to vary with a linear transmural variation from 60° at the endocardium to −60° at the epicardium in the LV wall.

### Closed‐loop circulatory model

**Figure 1:**
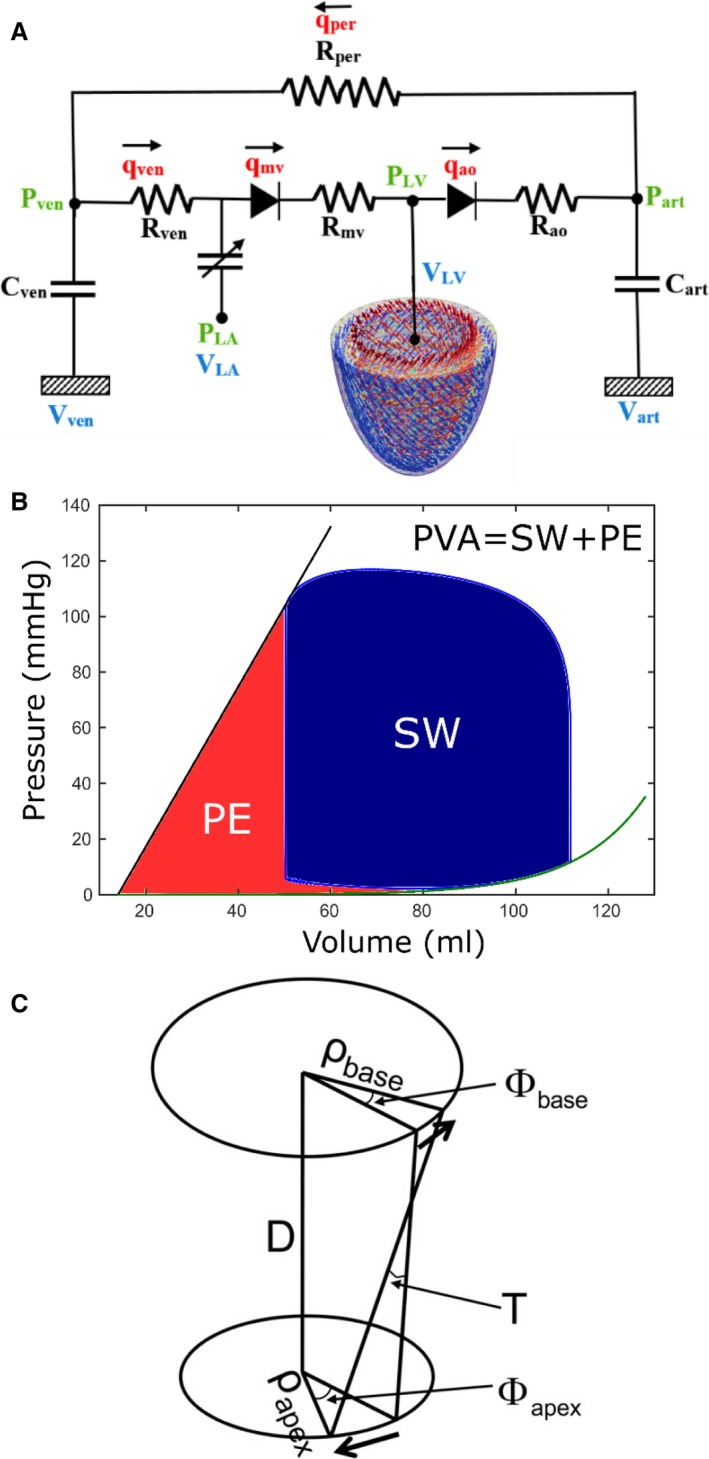
(A) Model schematic showing the coupling of LV FE model to a closed‐loop lumped parameter circulatory model, (B) Schematic showing the calculation of pressure**–**volume area (PVA), (C) Schematic showing the definition of LV torsion.

The LV FE model was coupled to a closed‐loop lumped parameter circulatory model (Fig. 1A). In this model, atrial contraction was simulated using a time varying elastance function. Details of the circulatory model and the parameter values are given in Appendix A. The initial volume states (*V*_ven_, *V*_art_) and the circulatory parameters were adjusted so that the steady‐state PV loop is consistent with that found in a typical normal human LV operating under resting conditions. Preload of the LV was varied by changing the venous volume *V*_ven,0_ to simulate vena cava occlusion. On the other hand, afterload was varied by altering the peripheral resistance *R*_per_ to simulate the constriction of vessels in the systemic vasculature. A steady‐state pressure**–**volume loop for each loading condition was established by running the simulation over several cardiac cycles, each with a cycle time of 900 msec (equivalent to 67 bpm).

### End‐systolic and end‐diastolic pressure**–**volume relationships

After obtaining PV loops at different preload and afterload, end‐systolic and end‐diastolic pressure**–**volume relationships (ESPVR and EDPVR, respectively) were obtained by performing regression on the following relationships:

(5a) $$P_{\mathrm{ESPVR}}\left(V\right)=E_{\mathrm{es}}\left(V-V_{0}\right)$$

(5b) $$P_{\mathrm{EDPVR}}\left(V\right)=A\left(e^{B\left(V-V_{0}\right)}-1\right)$$

where, $E_{\mathrm{es}}$ is the ESPVR slope, $V_{0}$ is the volume‐intercept, and *A*,* B* are parameters for the curvilinear EDPVR.

### Calculation of myocardial oxygen consumption

Details of calculating the local adenosine triphosphate (ATP) consumption during cross‐bridge cycling have been described previously (Hu et al. 2014) and can be found in Appendix B. The same methodology was applied here to quantify the ATP consumption rate at each integration point. Myocardial oxygen consumption was quantified using the total ATP consumption, which was computed by integrating the local ATP consumption rate over the entire LV through a complete cardiac cycle. The MVO_2_ at each loading case was related to the corresponding PVA, which is defined by the sum of the stroke work (i.e., external work done by the LV) and the end‐systolic potential energy (i.e., mechanical energy stored within elastic elements of the contractile proteins at the end of systole) (Fig. 1B).

### Calculation of myocardial strain

Regional three‐dimensional strains in the longitudinal, circumferential and radial directions were calculated using end‐diastole as the reference configuration. Specifically, myofiber stretch in these directions were expressed as:

(6) $$\lambda_{i}=\sqrt{e_{i}\cdot C\cdot e_{i}}$$

where, $C=F^{T}F$ is the right Cauchy‐Green deformation tensor, and **e**_*i*_ with *i *∈ *(l, c, r)* are the unit vectors in the longitudinal *l*, circumferential *c* and radial *r* directions respectively. The radial direction **e**_*r*_ is defined to be normal to the LV wall. The circumferential direction **e**_*c*_ is defined to be orthogonal to **e**_***r***_ and the apex‐base direction. Finally, the longitudinal direction, **e**_*l*_ is defined to be orthogonal to the both **e**_*r*_ and **e**_*c*_. This longitudinal direction is therefore tangential to the LV cavity wall surface. Different strain metrics, namely, the Biot, Green‐Lagrange strain and Euler‐Almansi strains were calculated using the following respective definitions:

(7a) $$\varepsilon_{i}=\lambda_{i}-1$$

(7b) $$E_{i}=\frac{1}{2}\left(\lambda_{i}^{2}-1\right)$$

(7c) $$e_{i}=\frac{1}{2}\left(1-\frac{1}{\lambda_{i}^{2}}\right)$$

### Calculation of left ventricular torsion

Left ventricular torsion T, which describes the amount of twisting the LV undergoes as it contracts, was calculated based on the relative rotation between the basal and the apical short axis slices (Fig. 1C) using the following (eq. 4):

(8) $$T=\frac{\left(\phi_{\mathrm{apex}}-\phi_{\mathrm{base}}\right)\left(\rho_{\mathrm{apex}}+\rho_{\mathrm{base}}\right)}{2D}\;$$

In equation (8), $\rho_{\mathrm{apex}}$ and $\rho_{\mathrm{base}}$ are the mean radius of curvature of the basal and apical slices, respectively, and, *D* is the distance between apical and basal slices. The angle of rotation at the apex *ϕ*_apex_ and base *ϕ*_base_ were calculated by tracking the motion of the nodal points at the apex and base respectively. Because the rotation varies across the LV wall, an average value of *T* was calculated using points on both the epicardial and endocardial surface.

## Results

### Isometric twitch behavior

**Figure 2:**
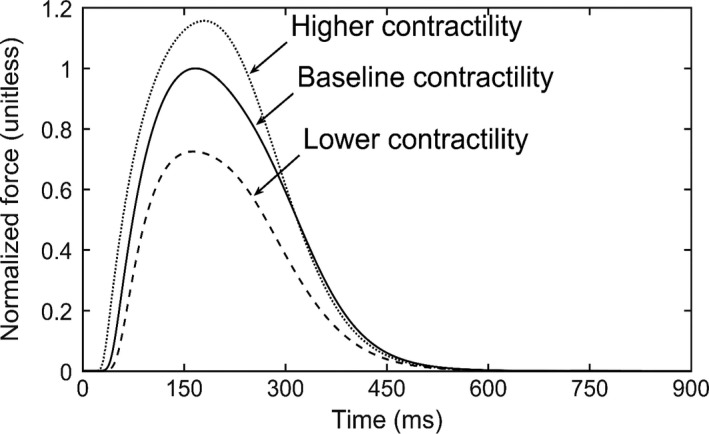
Isometric twitch profiles for different contractility cases. Force values were normalized by the maximum force of the baseline contractility case.

Isometric twitch profile computed using the adjusted rate constants (Table 1) shows that the time to peak value is ~170 msec (cf. to ~100 msec found in the original model using parameters calibrated using rat data) (Fig. 2). This value is consistent with the isometric twitch profile found in normal human (Land et al. 2017). On the other hand, scaling of the calcium transient in the electrophysiology model (Table 2) produces isometric twitch profiles that are self‐similar to each other.

### Pressure**–**volume loops

**Figure 3:**
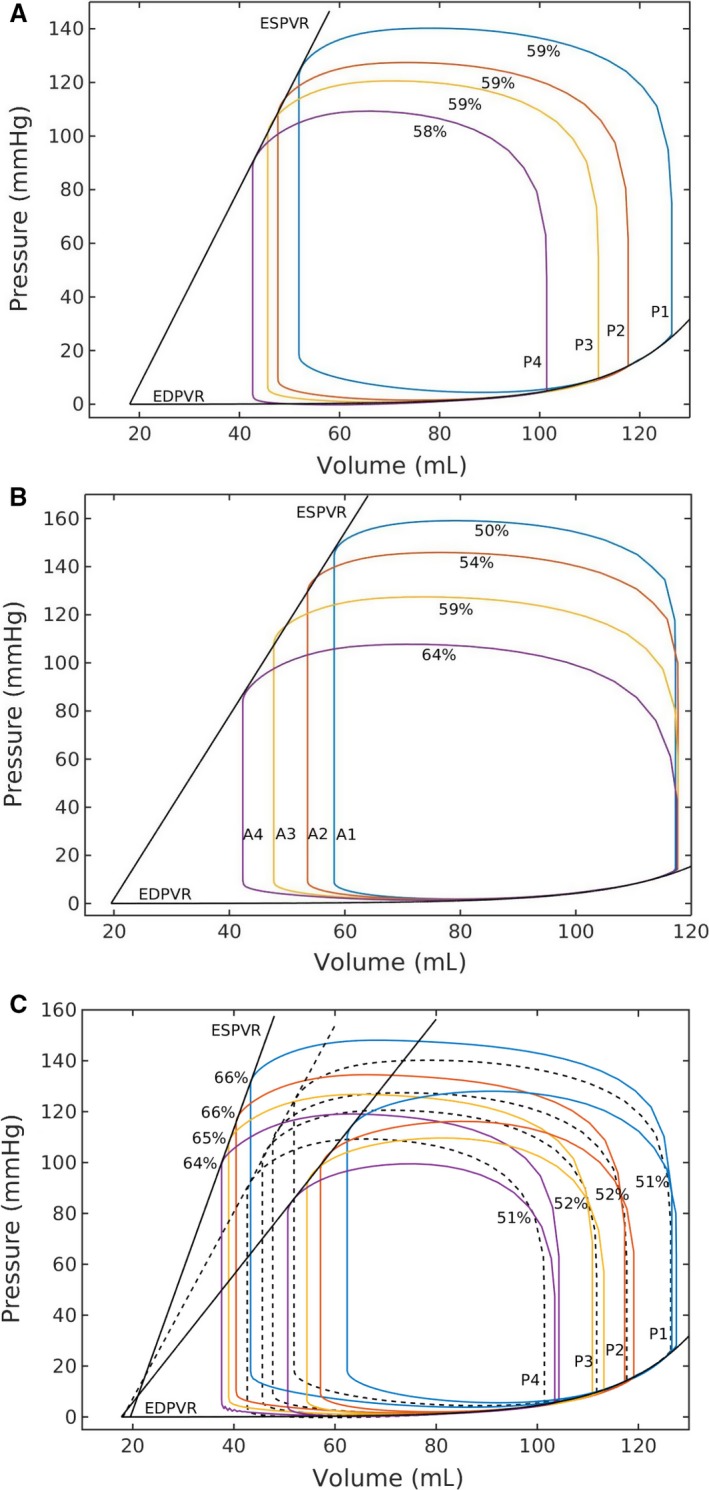
Effects on pressure**–**volume loop by due to a change in (A) preload at a constant afterload, (B) afterload at a constant preload, (C) contractility (solid) c.f. baseline (dotted), Values indicate corresponding ejection fraction.

Steady‐state PV loops of the LV under different loading conditions and contractilities were obtained from the FE model (Fig. 3). Increasing preload while maintaining a constant afterload resistance led to an increase in peak systolic pressure and shifted both the end‐systolic volume (ESV) and end‐diastolic volume (EDV) rightward toward larger volumes (Fig. 3A). Ejection fraction (EF), however, remained relatively constant between 58 and 59%. On the other hand, increasing afterload with a constant preload volume led to an increase in peak systolic pressure and ESV, with decreasing EF (Fig. 3B). In both cases (varying preload and afterload), the series of end‐systolic and end‐diastolic points derived from the PV loops produced a linear ESPVR and a curvilinear EDPVR. Also, an increase (or decrease) in contractility led to a corresponding increase (or decrease) in peak systolic pressure, EF, and the slope of the ESPVR (Fig. 3C).

Parameter values of *E*_es_, *V*_0_, *A* and *B* were calculated via regression analysis using equation (5a and b) applied to these data points (Table 3). The volume‐intercept *V*_0_ remained relatively constant in all the cases. On the other hand, the ESPVR slope *E*_es_ changed substantially (from the baseline case) only with varying contractility, and changed only a little in the case when afterload was varied.

**Table 3:** Parameters associated with ESPVR and EDPVR as found by the regression analysis

Parameter	Varying preload at			Varying afterload at baseline contractility
	Baseline contractility	Lower contractility	Higher contractility	
Slope of ESPVR, *E*_es_ (mmHg/mL)	3.67	2.51	5.54	3.81
Volume axis intercept, *V*_0_ (mL)	18.0	17.7	19.5	19.5
Scaling factor for EDPVR, *A* (mmHg)	0.021	0.021	0.021	0.021
Exponent for EDPVR, *B* (mL^−1^)	0.065	0.065	0.065	0.065

### MVO_2_–PVA relationships

**Figure 4:**
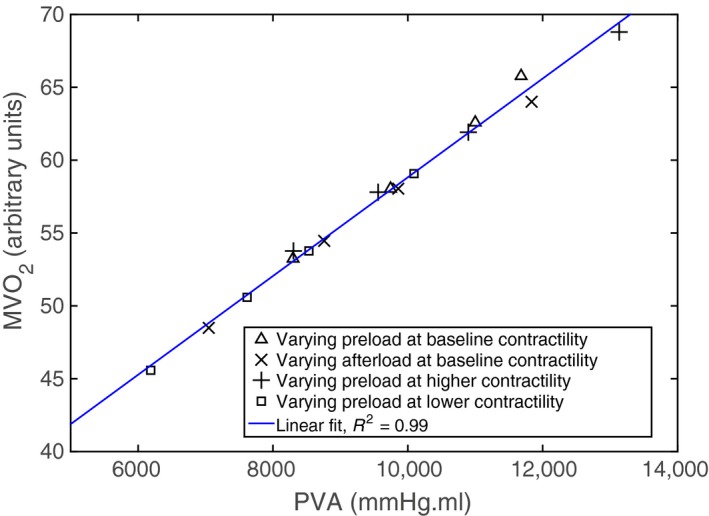
Myocardial oxygen consumption (MVO_2_) versus pressure**–**volume area (PVA) relationship predicted by the model. Data points are calculated at different preload, afterload and contractilites.

The relationship between PVA and the total LV ATP consumption in a cardiac cycle that is directly related to MVO_2_ were computed for different loading conditions and contractilities. The MVO_2_–PVA data calculated in all the cases having different preload, afterload and contractilities clustered around a straight line (Fig. 4). Regression performed on the data showed that the MVO_2_–PVA relationship is linear (*R* ^2^ = 0.99).

### Myocardial strain

**Figure 5:**
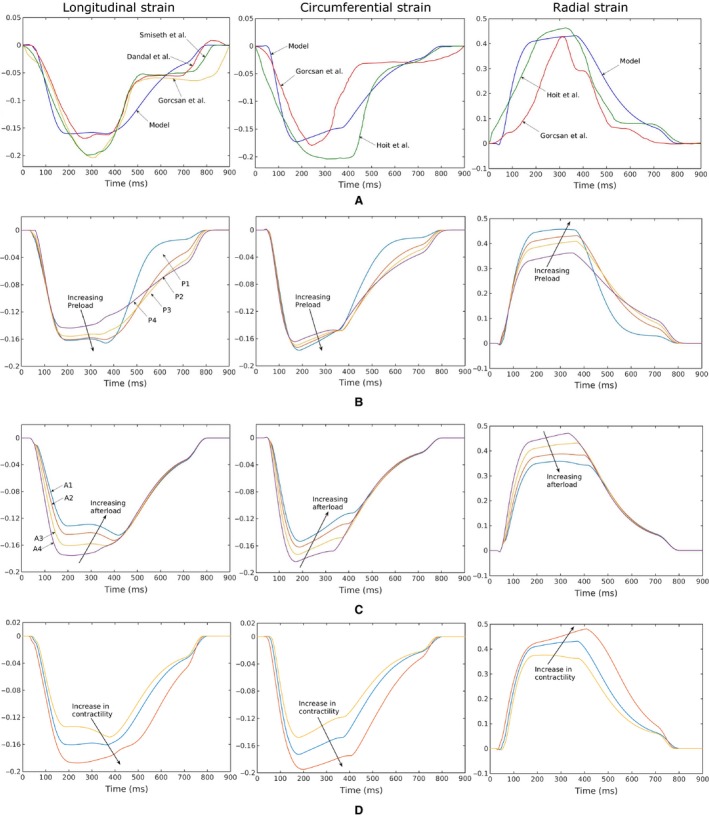
Longitudinal (first column), circumferential (second column), radial (third column) strain‐time profiles. (A) Comparison of the model predictions with previously published in vivo 2D STE measurements (Dandel et al. 2009; Gorcsan and Tanaka 2011; Hoit 2011). Strain‐time profiles predicted by the model with, (B) different preload (with constant afterload), (C) different afterload (with constant preload) and, (D) different contractility for a representative case (case P2 shown in Fig. 4A and C).

Longitudinal, circumferential and radial strain‐time profiles computed for different loading conditions and contractilities were compared to those measured in normal humans using speckle tracking echocardiography (STE) (Dandel et al. 2009; Gorcsan and Tanaka 2011; Hoit 2011) (Fig. 5). Under all loading conditions, the predicted strain‐time profiles were consistent with measurements in humans. Specifically, comparable features include a time‐to‐peak strain of about 200 msec during systole and a rapid change in strain at late diastole (~ 700 msec) arising from the contraction of left‐atrium (i.e. “atrial kick”). Peak strain values predicted by the model (~−16%, −18% and 40% in longitudinal, circumferential, and radial directions) were also comparable to those measured in normal humans.

Generally, peak circumferential and longitudinal strains were less sensitive to the loading conditions than radial strain. Varying preload filling pressure (by ~20 mmHg) at baseline contractility led to little change in the peak longitudinal strain (~2% absolute) or circumferential strain (~1% absolute). Peak radial strain, however, was substantially increased (~10% absolute) with increasing preload. On the other hand, peak strains were slightly more sensitive to variations in afterload than preload, where a 50 mmHg increase in afterload pressure was associated with reduced peak longitudinal, circumferential and radial strain of 4%, 3%, and 12% (absolute) respectively. An increase in contractility (with respect to baseline) led to an increase in the peak values of longitudinal (2.7% absolute), circumferential (2.2% absolute), and radial (5% absolute) strain with similar strain‐time characteristics. Similarly, the peak values of longitudinal (1.5% absolute), circumferential (2.5% absolute), and radial (6% absolute) strain decreased with a decrease in contractility with no change in strain‐time characteristics.

**Figure 6:**
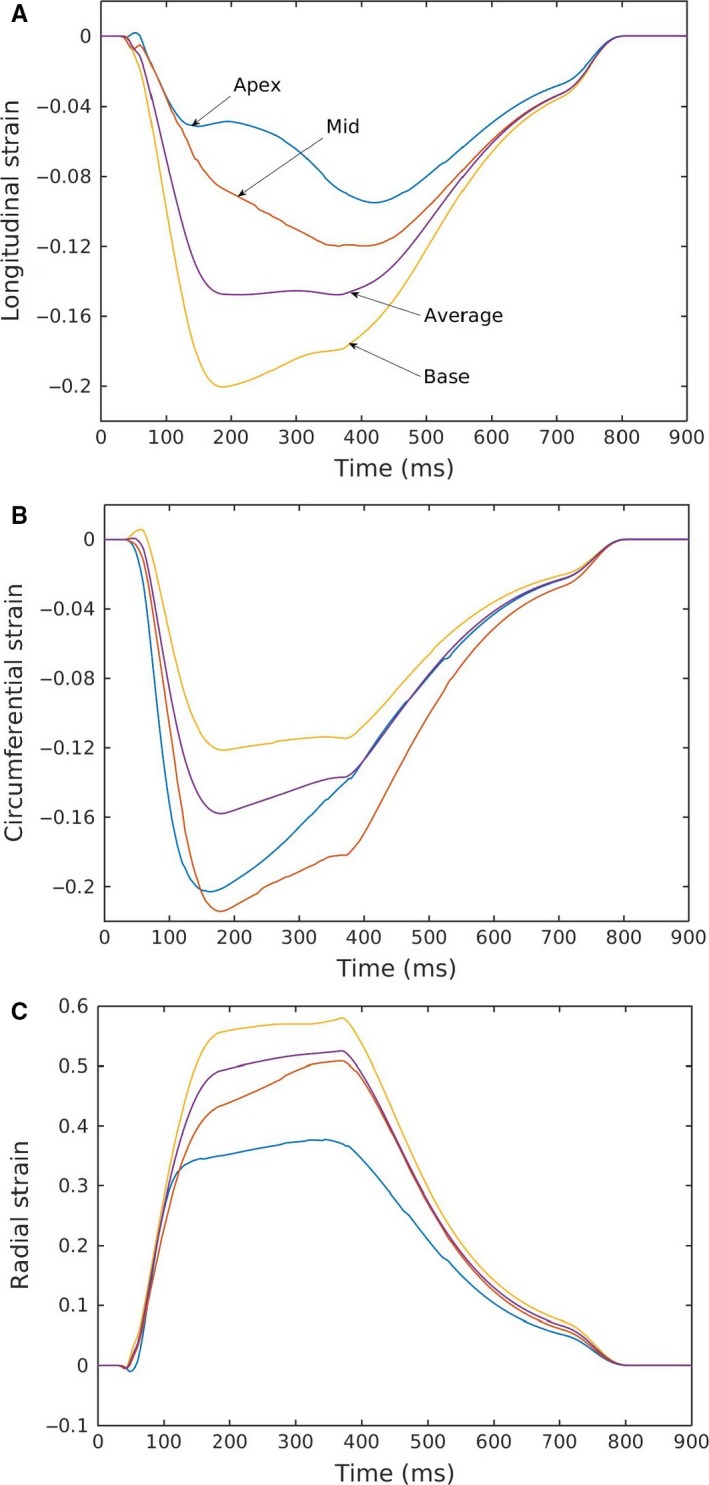
Regional variation in (A) longitudinal, (B) circumferential, and (C) radial strain profiles for a specific case corresponding with normal hemodynamic conditions (case P2, Fig. 4A).

Regional variation in strain profiles under different loading conditions followed a consistent pattern, shown in a representative case in Figure 6. Longitudinal strain was highest at the basal region and lowest at the apical region with a difference of about 10% (absolute). The same pattern was also found in the radial strain, where the difference between the highest strain (at the basal region) and the lowest strain (at the apical region) was about 20%. Regional variation in the circumferential strain was comparatively similar to the longitudinal strain, about 10%, with the highest value found at the mid‐LV and the lowest value found at the basal region.

**Figure 7:**
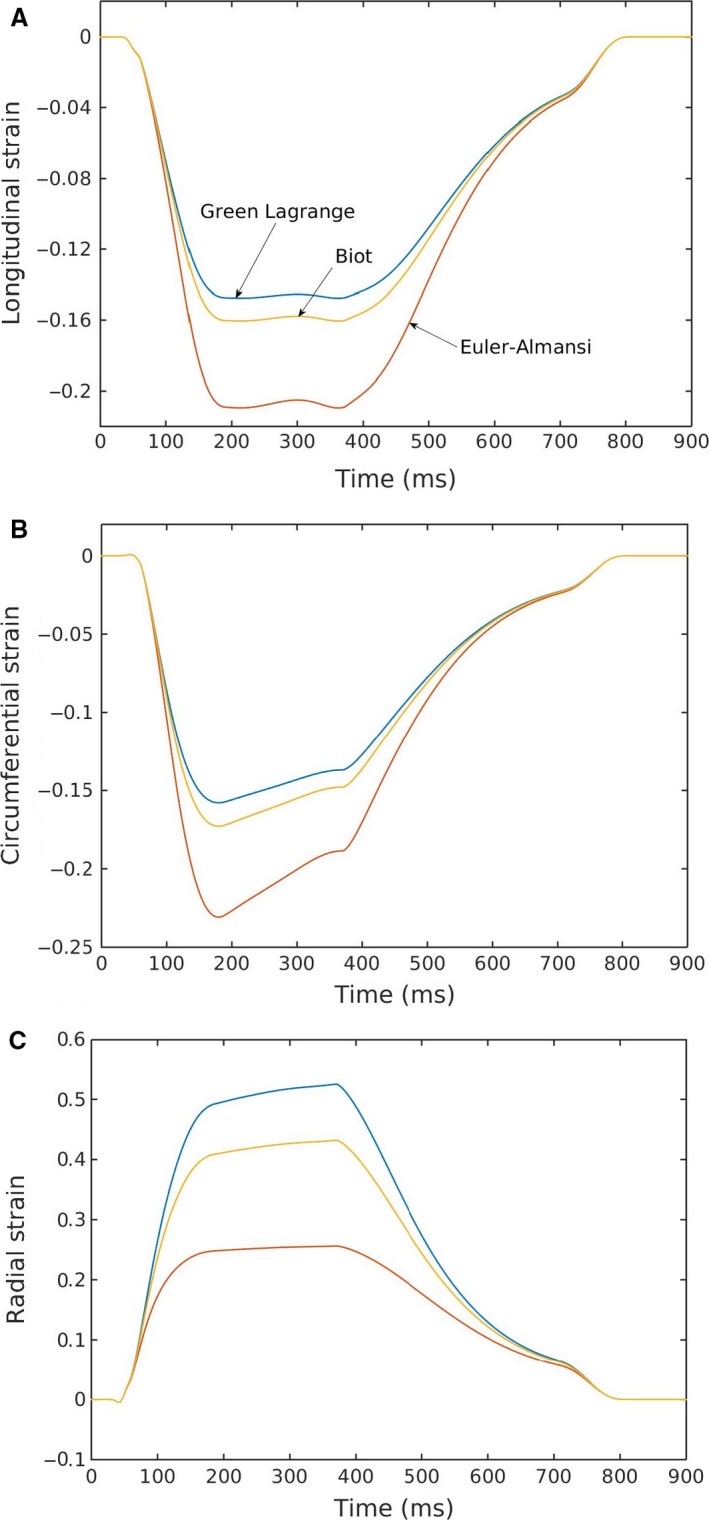
(A) Longitudinal, (B) circumferential, (C) radial strain profiles calculated using different strain definitions.

Using different strain definitions can lead to a substantial variation in the reported strain values (Fig. 7). Longitudinal and circumferential strains computed using the Euler‐Almansi definition were the largest followed by those computed using the Biot and Green‐Lagrange definitions. The reverse was found in the radial strain component, in which strains calculated using the Green‐Lagrange definition were the largest. The difference between these various strain definitions can be as large as ~27%, ~6%, and ~7% in the radial, longitudinal, and circumferential directions respectively.

### LV torsion

**Figure 8:**
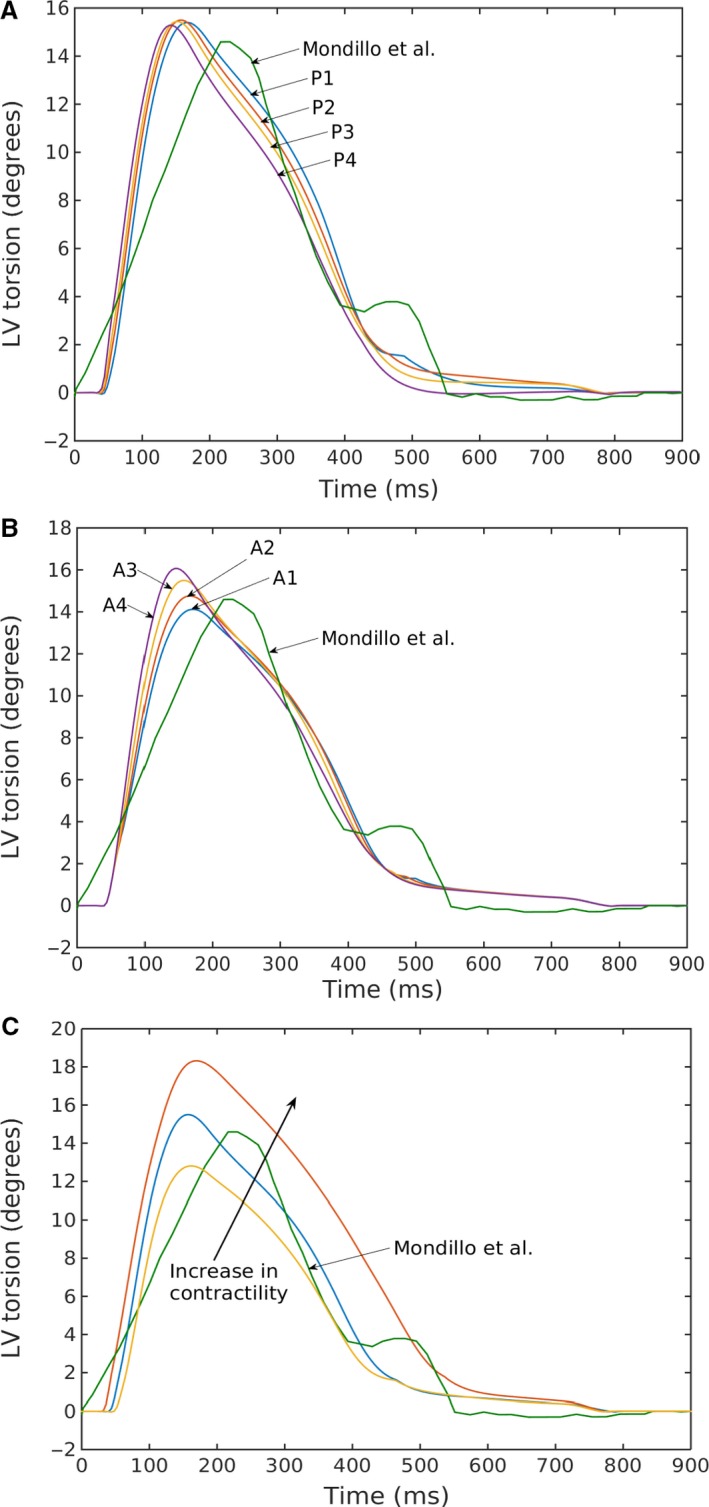
Left ventricular torsion for varying (A) preload, (B) afterload, and (C) contractility (case P2 in Fig. 3A and C) compared with echocardiographic measurements by Mondillo et al. (2011).

The time‐variation in LV torsion over a cardiac cycle was compared to the measurements made in normal human using echocardiography (Fig. 8) (Mondillo et al. 2011). Our model prediction of the peak LV torsion was close to that found in normal humans (~15°). Finally, LV torsion was also found to be relatively independent of the loading conditions. Similar to the effects of contractility on strains, a change in contractility (increase or decrease) led to a corresponding change in the peak value of LV torsion.

## Discussion

Finite element models simulating the LV mechanical behavior during a cardiac cycle have been developed using a variety of active contraction models (Kerckhoffs et al. 2007; Niederer and Smith 2009; Gurev et al. 2010, 2015). To the best of our knowledge, however, there have been no rigorous attempts to show that the models are able to simultaneously reproduce key organ–level physiology measured in the intact heart, specifically, (1) linear, load‐independent ESPVRs generated from PV loops of different loading conditions, (2) a linear, load‐independent MVO_2_ – PVA relationship, and (3) a consistent strain‐time profile. Here, we have filled this void and showed that a LV FE model based on the active contraction descriptor of Rice et al. (2008), with appropriate adjustment of model parameters, was able to reproduce these measured physiological features when coupled to a closed‐loop lumped parameter circulatory model.

Specifically, we demonstrated that both the slope and volume‐intercept of the linear ESPVR generated by varying preload and afterload are close to one another. The fitted values of *E*_es_ and *V*_0_ are also comparable to those measured in humans (McKay et al. 1986). Statistical analysis performed on the regressed values of *E*_es_ (Table 3) shows that the difference in values obtained for the varying preload and afterload cases (at the same baseline contractility) is not significant (*P* value = 0.57, 95% confidence interval). When contractility is varied (increased or decreased), however, the change in *E*_es_ (with respect to the baseline contractility cases) becomes significant (*P* value = 0.003, 95% confidence interval). As opposed to using pure phenomenological descriptors of active contraction (Kerckhoffs et al. 2007), MVO_2_ of the LV can be estimated here using the 4‐state active contraction descriptor by calculating the total ATP consumption required to uncouple the actin‐myosin bonds over a cardiac cycle, a reasonable estimate as approximately 90% of ATP generation is derived from aerobic metabolism. As MVO_2_ was estimated here solely from the cellular‐scale quantities in the 4‐state active contraction descriptor, correlating this calculated MVO_2_ to PVA, an organ‐scale quantity, serves as a rigorous and independent validation for the multiscale model. Consistent with the physiological measurements across species (Suga et al. 1981; Burkhoff et al. 1991; Wannenburg et al. 1992), our model predicts a linear relationship between MVO_2_ and PVA with varying preload, afterload and contractility.

In terms of deformation, our model predicted LV torsion as well as circumferential, longitudinal and radial strain‐time profiles that are agreeable with measurements from studies using echocardiography (Notomi et al. 2005; Dandel et al. 2009; Gorcsan and Tanaka 2011; Hoit 2011; Mondillo et al. 2011). Although these measurements showed significant variability, our model was able to reproduce key features, such as similar peak strains, time‐to‐peak‐strain as well as the rapid change in strain during atrial contraction at late‐diastole.

Using our model, we also compared the strains calculated using different definitions, namely, the Green‐Lagrange, Biot and Euler‐Almansi strain definitions. We showed that the difference could be as large as 27% in the radial direction and 6% in the longitudinal and circumferential directions when comparing between strains calculated using the Green‐Lagrange and Euler‐Almansi definitions. Circumferential and longitudinal strains computed using the Euler‐Almansi definition are larger than those calculated using the Green‐Lagrange definition whereas the opposite is true for radial strain (Fig. 7). This is consistent with the fact that the normalizing reference length used in the Green‐Lagrange definition is based on the ED configuration, at which length segments are at their longest in the circumferential and longitudinal directions and shortest in the radial direction. While most magnetic resonance (MR) based studies are explicit about the strain definition with Green‐Lagrange strain being the most commonly used metric (Moore et al. 2000; Lin et al. 2016), echo‐based studies are less clear concerning the type of metric used in computing myocardial strains. A number of echo‐based studies have described Lagrangian and Eulerian strain (Dandel et al. 2009; Smiseth et al. 2015). The definition of Lagrangian strain (*ε* = ∆*L*/*L* = *λ*−1) in those studies, however, is more commonly referred to as the Biot strain by the continuum mechanics community, and differs from the Green‐Lagrange strain that is frequently used in MR studies. The substantial disparity in strain computed using these two metrics (especially in computing radial strain) underscore the importance of using a consistent strain metric when comparing strain between different imaging modalities or between simulations and experiments.

We have also investigated the effects of preload and afterload on myocardial strains, and have showed that the strains are sensitive to changes in loading conditions at a fixed LV contractility. The radial strain is found to be the most sensitive, and all three strains are found to be sensitive to changes in afterload resistance that translated to relatively large changes in the peak LV pressures and EF. This result underscores the importance of not equating an evaluation of strains to an evaluation of myocardial contractility (Reichek 2013), especially when myocardial strains are increasingly used in diagnosing heart diseases such as myocardial ischemia, heart failure with preserved ejection fraction (HFpEF) and mechanical dyssynchrony (Dandel et al. 2009; Smiseth et al. 2015).

Particularly for HFpEF, a recent clinical study (Kraigher‐Krainer et al. 2014) has shown that the global longitudinal and circumferential strains are impaired in this patient population, and are about 5% points lower than those measured in normal humans (longitudinal: 20% vs. 14.6% absolute; circumferential: 27.1% vs. 22.9%, absolute). Stratifying HFpEF patients into categories with different ranges of LV EF, that study also showed a positive correlation between LV EF and both longitudinal and circumferential strains. A similar positive correlation between LV EF and the strains can also be found from our simulation results with varying afterload. In our simulation, the ESP ranges between 105 and 140 mmHg, which is equivalent to a systolic blood pressure range of 117–156 mmHg based on the empirical formula ESP = 0.9 × SBP (Kelly et al. 1992). Systolic blood pressure measured in the clinical study was only slightly higher in HFpEF patients (90% are hypertensive) than the normal subjects (136 vs. 130 mmHg). Our study therefore suggests that an increase in afterload may contribute, at least partially, to the decrease in longitudinal and circumferential strains found in HFpEF compared to normals. More study is clearly needed to separate the effects of a higher afterload from that of a decrease in contractility in contributing to the reduced longitudinal and circumferential strains in HFpEF patients.

### Model limitations

There are some limitations associated with the model. First, an idealized prolate ellipsoid was used to describe the LV geometry, which ignores any asymmetrical geometrical differences. Second, the model assumes that the LV contracts homogeneously and neglects any regional activation patterns. Third, a rule‐based myofiber orientation was prescribed in this model, in which the myofiber helix angle varied linearly in the transmural direction from endocardium to epicardium. The myofiber orientation, in reality, may be more complex. Last, mechanical effects associated with the right ventricle (RV) and pulmonary circulation was neglected. Although cavity pressure in the RV is substantially lower than the LV, its presence may, nevertheless, affects the LV mechanics through the septum.

## Conclusions

A LV FE model that is driven by a cell‐based descriptor of cross‐bridge cycling has been methodically validated against well‐established organ‐level physiological behaviors. The model parameters were adjusted appropriately to confirm that it performs in a manner consistent with experimental observations on the impact of preload, afterload, and contractility on the ESPVR and MVO_2_–PVA relationships. Furthermore, the model can reproduce time‐strain profiles that are consistent with physiological measurements. The model will be used in future studies to address important unanswered questions about human heart diseases that cannot be resolved through experimentation, such as whether changes in longitudinal strain as observed in HFpEF patients reflects a change in myocardial contractility or is simply a reflection of alterations in load.

## Appendix A Circulatory Model

### Closed‐Loop Circulation

For blood vessels, pressure is found from the following equations,

(A1) $$P_{\mathrm{art}}=\frac{V_{\mathrm{art}}-V_{\mathrm{art},0}}{C_{\mathrm{art}}}$$

(A2) $$P_{\mathrm{ven}}=\frac{V_{\mathrm{ven}}-V_{\mathrm{ven},0}}{C_{\mathrm{ven}}}$$

where, *V*_art,0_ and *V*_ven,0_ are resting volumes of the blood vessels and both are constants.

Flowrates at different sections are given by,

(A3) $$q_{\mathrm{ao}}=\left\{\begin{array}{cc} \frac{P_{\mathrm{LV}}-P_{\mathrm{art}}}{R_{\mathrm{ao}}} & \text{when},P_{\mathrm{LV}}\geq P_{\mathrm{art}}\\ 0 & \text{when},P_{\mathrm{LV}}<P_{\mathrm{art}} \end{array}\right.$$

(A4) $$q_{\mathrm{per}}=\frac{P_{\mathrm{art}}-P_{\mathrm{ven}}}{R_{\mathrm{per}}}$$

(A5) $$q_{\mathrm{ven}}=\frac{P_{\mathrm{ven}}-P_{\mathrm{LA}}}{R_{\mathrm{ven}}}$$

(A6) $$q_{\mathrm{mv}}=\left\{\begin{array}{cc} \frac{P_{\mathrm{LA}}-P_{\mathrm{LV}}}{R_{\mathrm{mv}}} & \text{when},P_{\mathrm{LA}}\geq P_{\mathrm{LV}}\\ 0 & \text{when},P_{\mathrm{LA}}<P_{\mathrm{LV}} \end{array}\right.$$

Finally, the rate of change in volume for the cardiac chambers (LV and LA) and blood vessels (Aorta and vena cava) can be related to the flowrates by the following equations,

(A7) $$\frac{dV_{\mathrm{LA}}}{dt}=q_{\mathrm{ven}}-q_{\mathrm{mv}}$$

(A8) $$\frac{dV_{\mathrm{LV}}}{dt}=q_{\mathrm{mv}}-q_{\mathrm{ao}}$$

(A9) $$\frac{dV_{\mathrm{art}}}{dt}=q_{\mathrm{ao}}-q_{\mathrm{per}}$$

(A10) $$\frac{dV_{\mathrm{ven}}}{dt}=q_{\mathrm{per}}-q_{\mathrm{ven}}$$

**Table A1:** Normal parameter values of the circulatory model

Parameter	Unit	Values
Aortic valve resistance, *R*_ao_	Pa·msec·mL^−1^	5500
Peripheral resistance, *R*_per_	Pa·msec·mL^−1^	14,0000
Venous resistance, *R*_ven_	Pa·msec·mL^−1^	2000
Mitral valve resistance, *R*_mv_	Pa·msec·mL^−1^	2500
Aortic compliance, *C*_art_	mL·Pa	0.014
Venous compliance, *C*_ven_	mL·Pa	0.3
Resting volume for artery, *V*_art,0_	mL	580
Resting volume for vein, *V*_ven,0_	mL	3300

### Time Varying Elastance for Left Atrium

Time‐varying elastance model was used to describe atrial contraction by relating the instantaneous atrial pressure *P*_LA_(*t*) to the instantaneous atrial volume *V*_LA_(*t*) using the following equations

(A11) $$P_{\mathrm{LA}}\left(t\right)=e\left(t\right)P_{\mathrm{es},\mathrm{LA}}\left(V_{\mathrm{LA}}\right)+\left(1-e\left(t\right)\right)P_{\mathrm{ed},\mathrm{LA}}\left(V_{\mathrm{LA}}\right)$$

where,

(A12) $$P_{\mathrm{es},\mathrm{LA}}\left(V_{\mathrm{LA}}\right)=E_{\mathrm{es},\mathrm{LA}}\left(V_{\mathrm{LA}}-V_{0,\mathrm{LA}}\right)$$

(A13) $$P_{\mathrm{ed},\mathrm{LA}}\left(V_{\mathrm{LA}}\right)=A_{\mathrm{LA}}\left(e^{B_{\mathrm{LA}}\left(V_{\mathrm{LA}}-V_{0,\mathrm{LA}}\right)}-1\right)$$

and,

(A14) $$e\left(t\right)=\left\{\begin{array}{cc} \frac{1}{2}\left(\sin\left[\left(\frac{\pi }{T_{\max}}\right)t-\frac{\pi }{2}\right]+1\right); & 0<t\leq 3/2T_{\max}\\ \frac{1}{2}e^{-\left(t-3/2T_{\max}\right)/\tau }; & t>3/2T_{\max} \end{array}\right.$$

In the above, *E*_es,LA_ is the end‐systolic elastance, *V*_0,LA_ is the volume axis intercept of the ESPVR, and both *A*_LA_ and *B*_LA_ are parameters of the EDPVR. The driving function $e\left(t\right)$ is described in Equation (A14), where *T*_max_ is the point of maximal chamber elastance, *τ* is the time constant of relaxation and *t* is the time during the cardiac cycle. The values of *E*_es,LA_, *V*_0,LA_, *A*_LA_, *B*_LA_, *T*_max_, and *τ* that are used in this model are listed in Table A2.

**Table A2:** Parameters of time varying elastance model for left atrium

Parameter	Unit	Values
End‐systolic elastance, *E*_es,LA_	Pa/mL	60
Volume axis intercept, *V*_0,LA_	mL	10
Scaling factor for EDPVR, *A*_LA_	Pa	58.67
Exponent for EDPVR, *B*_LA_	mL^−1^	0.049
Time to end‐systole, *T*_max_	msec	200
Time constant of relaxation, *τ*	msec	25

## Appendix B Calculation of ATP from Rice Model

The total ATP consumption was determined by integrating the ATP consumption rate computed at each integration point over the LV wall volume and over time for the entire cardiac cycle (900 msec in duration). The local ATP consumption rate was calculated from the Rice model (Rice et al. 2008) by multiplying the cross‐bridge detachment rate by the single‐overlap fraction of the thick filament and the probability that the cross‐bridge is in the postrotated force‐generating state. In mathematical terms,

(B1) $$\text{ATP}=g_{xbT}XB_{\mathrm{PostR}}SOVF_{\mathrm{thick}}\left(x\right)$$

where, *g*_*xbT*_ is the cross‐bridge detachment rate, *XB*_PostR_ is the probability that the cross‐bridge is in the postrotated force‐generating state, $SOVF_{\mathrm{thick}}\left(x\right)$ is the single‐overlap function for the thick filament, *x* is the sarcomere length.
